# IL-33 promotes efferocytosis by peritoneal macrophages by a mechanism associated with rapid granulocyte IL-13 production

**DOI:** 10.3389/fimmu.2026.1802300

**Published:** 2026-07-22

**Authors:** Stephanie A. Legere, Roopa Hebbandi Nanjundappa, Dihia Meghnem, Ian D. Haidl, Alexander Edgar, Qianni Hu, Jean-François Légaré, Jean S. Marshall

**Affiliations:** 1 Department of Microbiology and Immunology, Dalhousie University, Halifax, NS, Canada; 2 Department of Pathology, Dalhousie University, Halifax, NS, Canada; 3 Dalhousie Medicine New Brunswick, Saint John, NB, Canada; 4 New Brunswick Heart Centre, Saint John, NB, Canada

**Keywords:** alarmin cytokine, cytokines, eosinophil, inflammation, inflammation resolution, mast cell, MerTK, phagocytosis

## Abstract

Resolution of inflammation is an active process that requires efferocytosis, the engulfment of apoptotic cells by macrophages, mediated by receptors such as MerTK. IL-33 is an alarmin that initiates type 2 immune responses, including increased production of IL-13, which promotes MerTK expression. The ability of IL-33 to promote efferocytosis *in vivo* was examined. Intraperitoneal administration of IL-33 to mice increased local MerTK^+^ macrophage numbers within 48h. MerTK^+^ macrophages were not similarly induced by free mitochondria, an alternative cell damage associated signal. Efferocytotic activity was increased rapidly in response to apoptotic thymocytes in IL-33-treated mice. The established inducer of MerTK expression, IL-13, was detected in peritoneal lavage fluid shortly after IL-33 administration. Peritoneal eosinophils expressed the IL-33 receptor and demonstrated both intracellular IL-13 by flow cytometry and significantly increased *Il13* transcript expression following IL-33 treatment. In contrast, neither elevated IL-13 expression nor IL-13 protein secretion was observed in peritoneal lymphocyte populations within the first 6 hours after IL-33 administration. Primary cultures of bone marrow-derived mouse mast cells and eosinophils demonstrated IL-13 protein responses to IL-33 administration within 6 h. Mast cell-deficient Cpa3-Cre; Mcl-1^fl/fl^ mice had significantly reduced IL-13 levels in the peritoneal cavity 3 hours after IL-33 administration when compared with mast cell-containing littermates. In contrast, IL-33-treated eosinophil-deficient ΔdblGATA mice had similar levels of IL-13 at this time point as wild type controls. These data demonstrate that IL-33 promotes MerTK expression, critical for efferocytosis by macrophages, by a process associated with an early rapid local increase in IL-13 production to which mast cells are a substantial early contributor. These findings contribute to our understanding of clinical situations where elevated soluble IL-33 receptor (sST2) and/or lower mast cell numbers are associated with worse clinical outcome.

## Introduction

Inflammation is crucial for responding to infection and tissue trauma and, when properly regulated, is followed by restoration of tissue homeostasis (1, 2). Resident immune cells generate multiple mediators such as cytokines, chemokines, and eicosanoids, to promote polymorphonuclear cell recruitment and elimination of pathogens or cell debris (3). Proper orchestration of inflammation resolution is key, as aberrant mediator or resolution processes can lead to chronic inflammation that can result in fibrosis, cancer and autoimmune disease (4–6). Debris removal from inflamed tissues must occur to limit further inflammatory mediator production (1, 2). Neutrophils are recruited to sites of tissue damage and undergo apoptosis *in situ* within 24-48h (7). Their removal is critical for inflammation resolution, as secondary necrotic cells perpetuate tissue damage (8, 9). Efferocytosis, macrophage- mediated engulfment of apoptotic cells (10) is a central linking step in this resolution process, mechanistically distinct from phagocytosis (11, 12). At sites of damage or inflammation, macrophages engulf apoptotic cells via recognition of “eat me” signals such as phosphatidylserine on the target cell surface (13). Such signals interact with several macrophage receptors including the efferocytosis receptor, Myeloid-epithelial-reproductive Tyrosine Kinase (MerTK) (14), a receptor tyrosine kinase of the TYRO3, AXL and Mer (TAM) receptor family (15). Resolution of inflammation processes in several sterile inflammatory and tissue injury settings have been shown to involve MerTK (16–19).The surface expression of MerTK on macrophages is induced by multiple signals, including IL-13 stimulation in the presence of apoptotic cells (20–22). *Mertk-/-* mice have marked accumulation of apoptotic cells in their tissues due to the absence of efferocytosis by macrophages (23) and eventually develop spontaneous autoimmune disease as a result (24, 25). Through a separate mechanism in some tissue settings, IL-13 has been shown to induce macrophage IL-10 production and enhance efferocytosis through a Vav1- and Rac1-dependent pathway that facilitates apoptotic cell engulfment (21). Regulatory T cells and innate lymphoid cells have previously been implicated as important sources of IL-13 in this process (21, 26). However, within the peritoneum, multiple other cell types can produce IL-13 in response to alarmin stimulation including eosinophils and mast cells (9).

IL-33 is known as a dual function cytokine. It is released upon cell death or nuclear damage by fibroblasts, endothelial cells, epithelial cells, and other stromal cells (27). Once in the extracellular environment, IL-33 acts to initiate type 2 inflammatory responses via signaling through its cognate receptor complex of IL1RL1 (ST2) and IL-1RAcP (IL-1-receptor Accessory Protein), which is expressed on mast cells (28), type 2 innate lymphoid cells (ILC2s) (29), eosinophils (30), CD4^+^ T regulatory (Treg) (31) and T helper 2 cells (Th2) (32). In this way, IL-33 functions both as a DAMP and alarmin, signaling through a cytokine receptor (33). IL-33 signaling results in the generation of several mediators, including IL-13 which has roles in type 2 inflammation and in tissue remodeling events (30–33). However, IL-33 can be rapidly degraded by proteolysis (34, 35) and oxidation (36) in the extracellular space, making its initial, microenvironmental responses most relevant. In this study, we sought to determine if IL-33 promotes efferocytosis in the peritoneal cavity which is rich in both resident macrophages and ST2 positive effector cells. We assessed changes in peritoneal leukocyte populations and the early sources of IL-13, in response to IL-33. Our data suggest a critical early role for mast cells in IL-13 production, with an additional contribution from eosinophils, during the initial IL-13 response to IL-33, with little initial role for lymphocyte derived IL-13 in this tissue site. Local IL-13 responses have been implicated in driving MerTK expression (20, 37) suggesting a role for resident immune cells in this process.

## Results

### IL-33 modifies populations of macrophages in the peritoneal cavity

**Figure 1:**
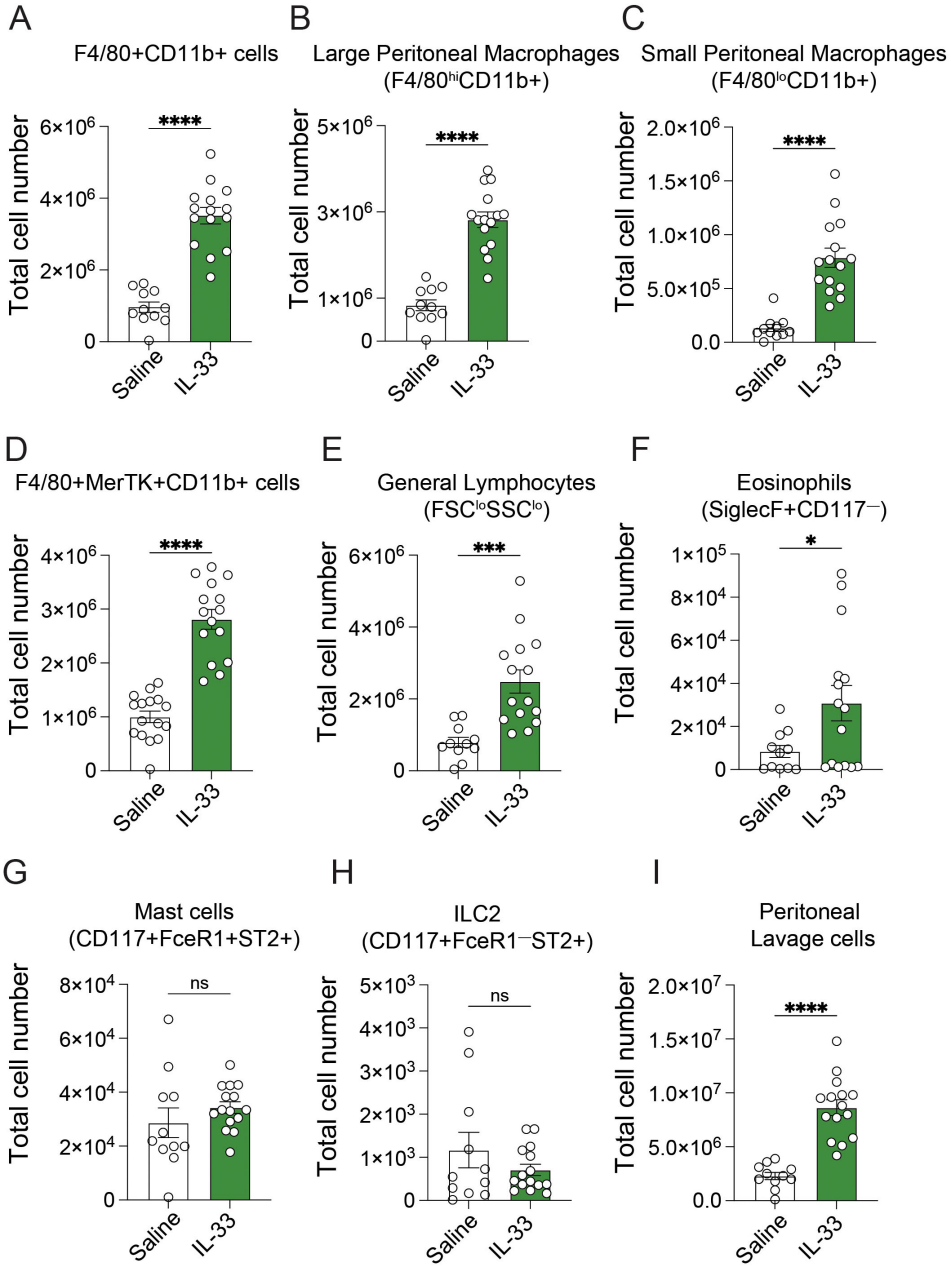
IL-33 increases macrophage and other leukocyte population in the peritoneal cavity. Female C57BL/6 mice were injected i.p. with 0.5μg IL-33 (n=15) or saline (n=11) and sacrificed 48 hours later. Leukocyte populations in the peritoneal cavity were assessed by flow cytometry, and total cell numbers are represented as mean ± SEM. IL-33 treatment significantly increased total F4/80^+^CD11b^+^ macrophages **(A)**, large peritoneal macrophages (LPM; F4/80^hi^CD11b^+^) **(B)**, small peritoneal macrophages (SPM; F4/80^lo^CD11b^+^) **(C)**, MerTK^+^ macrophage (F4/80^+^ MerTK^+^ CD11b^+^) **(D)**, general lymphocytes (FSC^lo^SSC^lo^) **(E)**, eosinophils (Siglec-F^+^CD117^-^) **(F)**, but did not significantly alter mast cells (CD117^+^FcϵRI^+^ST2^+^) **(G)** or ILC2s (CD117^+^FcϵRI^-^ST2^+^) **(H)**. Total peritoneal lavage cell numbers were also increased **(I)**. Statistical significance was determined by unpaired t-test. *p<0.05, ***p<0.001, ****p<0.0001; ns, not significant.

Leukocyte populations were assessed in the peritoneal cavities of both female and male mice at 48 hours post i.p. IL-33 injection via flow cytometry (gating strategy is shown in **Supplementary Figures S1A–C**). IL-33-treated female mice had increased overall F4/80^+^CD11b^+^ macrophage populations (**Figure 1A**) compared to saline treated controls. There are two distinct populations of macrophages reported at this site (38), F4/80^hi^CD11b^+^ large peritoneal macrophages (LPM; **Figure 1B**), and F4/80^lo^CD11b^+^ small peritoneal macrophages (SPM; **Figure 1C**). Both populations were significantly increased in number in response to IL-33 treatment. Total numbers of MerTK^+^ macrophages in the peritoneal cavity (F4/80^+^CD11b^+^MerTK^+^; **Figure 1D**) were increased at 48 h post IL-33 injection, as were MerTK^+^ LPMs (**Supplementary Figure S3B**), and SPMs (**Supplementary Figure S3C**).

Several leukocyte populations were increased in the peritoneal cavity after IL-33 treatment, including total lymphocytes (FSC^lo^SSC^lo^; **Figure 1E**), eosinophils (Siglec-F^+^CD117^-^; **Figure 1F**), neutrophils (Ly6C^low^Ly6G^+^; **Supplementary Figure S3G**) as reflected in increased total peritoneal lavage cells (**Figure 1I**). Inflammatory monocytes (Ly6C^+^CCR2^+^) were significantly increased in IL-33-treated mice compared to saline (**Supplementary Figure S3H**), while patrolling monocytes (Ly6C^lo^CX3CR1^+^) were not (**Supplementary Figure S3I**). Populations of mast cells (**Figure 1G**) and ILC2s (**Figure 1H**) were not significantly impacted, in terms of cell number, despite their known high expression of ST2 (39, 40). While overall numbers of macrophages and SPM populations were increased in male mice (**Supplementary Figure S2**), a more robust impact on leukocytes, and MerTK^+^ macrophages, was observed in female mice (**Supplementary Figure S3**). Therefore, female mice were used for the majority of further studies. As such, the functional conclusions are primarily supported in female mice.

### IL-33 enhances efferocytosis in the peritoneal cavity

**Figure 2:**
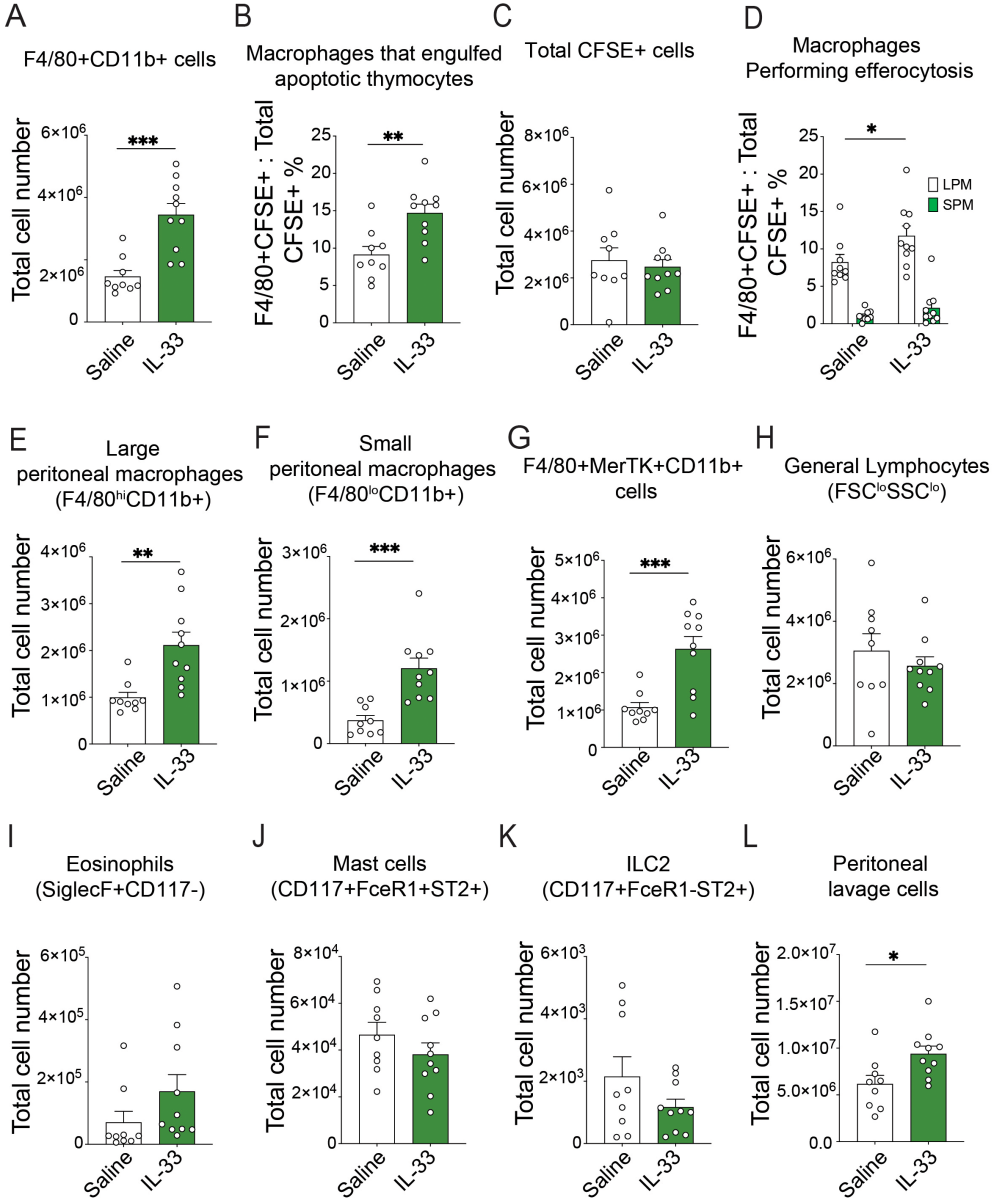
IL-33 increases efferocytosis and associated macrophage populations in the presence of apoptotic cells. Female C57BL/6 mice were injected i.p. with 0.5μg IL-33 (n=10) or saline (n=9) for 48h, followed by i.p. administration of 2.5 x 10^7^ CFSE-labeled apoptotic thymocytes (30-35% Annexin V^+^) 30 minutes prior to harvest. Total cell numbers are represented as mean ± SEM. Flow cytometry analysis quantified overall F4/80^+^CD11b^+^ macrophages **(A)**; percentage of macrophages containing apoptotic thymocytes (F4/80^+^CFSE^+^) as a measure of efferocytosis **(B)**; LPM and SPM subsets within F4/80^+^CFSE^+^ macrophages **(C)**; total CFSE^+^ cells in the peritoneal cavity **(D)**; LPM (F4/80^hi^CD11b^+^) **(E)**; SPM (F4/80^lo^CD11b^+^) **(F)**; MerTK^+^ macrophages (F4/80^+^MerTK^+^CD11b^+^) **(G)**; general lymphocytes (FSC^lo^SSC^lo^) **(H)**; eosinophils (SiglecF^+^CD117^—^) **(I)**; mast cells (CD117^+^FcϵRI^+^ST2^+^) **(J)**; ILC2s (CD117^+^FcϵRI^—^ST2^+^) **(K)**; and total peritoneal lavage cells **(L)**. Statistical significance was determined using an unpaired t-test. Data represent the combined results of three independent experiments. *p<0.05, **p<0.01, ***p<0.001.

To determine the impact of IL-33 administration on peritoneal efferocytosis, mice were treated i.p. with IL-33 or saline, followed 48 hours later by an i.p. injection of 2.5 x 10^7^ CFSE-labeled apoptotic thymocytes (30-35% apoptotic; **Supplementary Figure S1D**). After a further 30 minutes, peritoneal leukocyte populations were quantitated and efferocytosis was assessed. Peritoneal F4/80^+^CD11b^+^ macrophage numbers were increased after administration of IL-33 and apoptotic cells compared to apoptotic cells and saline (**Figure 2A**). Efferocytosis was significantly increased in the peritoneal cavity after IL-33 administration, as indicated by an increased percentage of F4/80^+^CFSE^+^ cells, i.e. macrophages that had engulfed thymocytes as a proportion of total CFSE^+^ cells (**Figure 2B**) using the gating strategy as shown (**Supplementary Figure S1E**). Normalization to total macrophage numbers did not reveal increased per-cell efferocytic capacity, indicating that this effect is primarily driven by increased macrophage abundance. The total numbers of CFSE^+^ cells in the peritoneal cavity were consistent between groups (**Figure 2C**). LPMs demonstrated significantly more efferocytosis in response to IL-33 and apoptotic cell injection compared to LPMs from mice treated with saline (control) in place of IL-33 (**Figure 2D**). SPMs did not significantly differ in their efferocytosis abilities with or without IL-33 treatment (**Figure 2D**). Both F4/80^hi^CD11b^+^ LPMs (**Figure 2E**), and F4/80^lo^CD11b^+^ SPM (**Figure 2F**), populations significantly increased in number in response to IL-33 treatment. The proportion of MerTK^+^ macrophages (**Figure 2G**) was consistently increased following IL-33 treatment compared to controls. However, numbers of lymphocytes (**Figure 2H**), and eosinophils (**Figure 2I**) were not impacted by the IL-33 and apoptotic cell treatment in the peritoneal cavity. Mast cell (**Figure 2J**) and ILC2 numbers (**Figure 2K**) also remained unchanged following addition of apoptotic cells. Increased overall numbers of peritoneal lavage cells were observed in IL-33 injected mice (**Figure 2L**), as also observed in **Figure 1**.

### Free mitochondria modify the response to IL-33 in the peritoneal cavity

**Figure 3:**
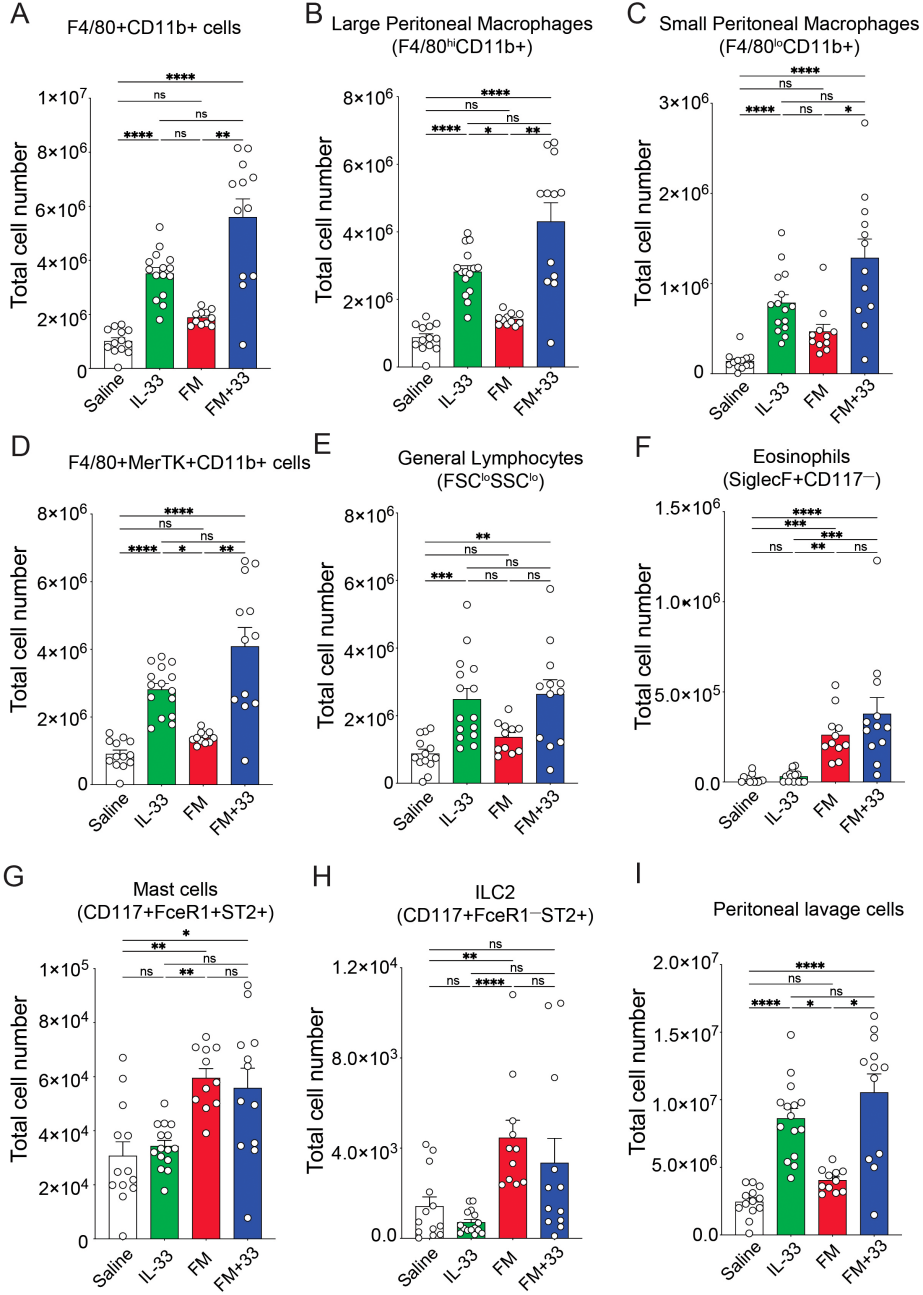
Free mitochondria do not induce MerTK^+^ macrophages in the peritoneal cavity but act synergistically with IL-33 to enhance some leukocyte population. Female C57BL/6 mice were injected i.p. with 50 μg of free mitochondria (FM, n=11, 0.5 μg IL-33 (n=15), both FM and IL-33 (FM + 33, n=12) or saline (n=13) for 48h prior to sacrifice. Leukocyte populations in the peritoneal cavity were assessed by flow cytometry and total cell numbers are shown as mean ± SEM. Panels show total F4/80^+^CD11b^+^ macrophages **(A)**, large peritoneal macrophages (LPM; F4/80^hi^CD11b^+^) **(B)**, small peritoneal macrophages (SPM; F4/80^lo^CD11b^+^) **(C)**, MerTK^+^ macrophages (F4/80^+^MerTK^+^CD11b^+^) **(D)**, general lymphocytes (FSC^lo^SSC^lo^) **(E)**, eosinophils (SiglecF^+^CD117^—^) **(F)**, mast cells (CD117^+^FcϵRI^+^ST2^+^) **(G)**, ILC2s (CD117^+^FcϵRI^—^ST2^+^) **(H)**, and total peritoneal lavage cells **(I)**. Total numbers are represented as mean ± SEM. Statistical significance was determined using one-way ANOVA. Data represent the combined results of three independent experiments. *p< 0.05, **p< 0.01, ***p< 0.001, ****p< 0.0001, ns, not significant.

IL-33 is one of many DAMPs and alarmins released by dead or dying cells during tissue damage or infection. Therefore, we considered the possibility that alternative DAMPs, independent of IL-1 family cytokines, may impact efferocytosis or modify the impact of IL-33. A common feature of necrotic cell death is the release of free mitochondria (FM) (41). As such, we sought to determine if such FM, which are associated with multiple DAMP signals, affect the MerTK^+^ macrophage profile in the peritoneal cavity. Mitochondria were isolated from murine liver and administered i.p. with or without IL-33–48 hours prior to harvest of peritoneal lavage. FM administration alone did not impact macrophage populations of interest or alter MerTK expression (**Figures 3A–D**). However, when FM were co-administered with IL-33, there was a trend towards increased macrophage numbers (**Figures 3A–D**), as well as lymphocytes (**Figure 3E**) and overall cell number in the peritoneal cavity (**Figure 3I**), although these did not reach statistical significance. FM administration alone led to a significantly increased populations of eosinophils (**Figure 3F**), mast cells (**Figure 3G**) and ILC2s (**Figure 3H**) in the peritoneal cavity. These data suggest a coordinated response between IL-33 and mitochondrial DAMPs as distinct immune signals, both associated with cell damage. These data indicate that mitochondrial DAMPs do not significantly enhance IL-33-mediated responses under these conditions.

### IL-33 induces IL-13 production in the peritoneal microenvironment

**Figure 4:**
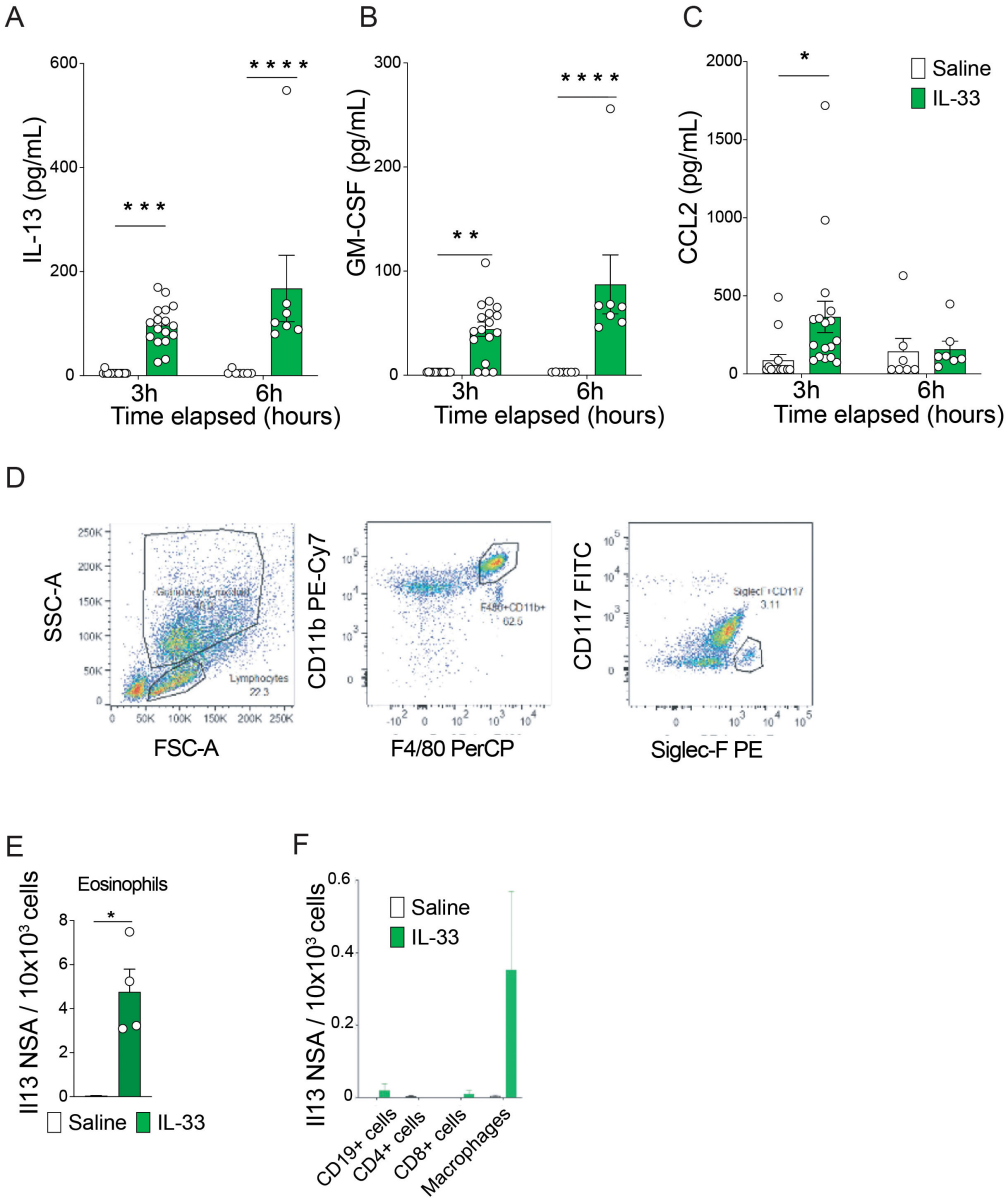
IL-33 induces IL-13, GM-CSF and CCL2 in the peritoneal cavity. Female C57BL/6 mice were injected i.p. with 0.5μg IL-33 or saline, and sacrificed 3h later (IL-33, n=17; saline, n=16, five separate experiments) or 6h (IL-33, n=7; saline, n=7; three separate experiments). Results are shown as the mean ± SEM. Peritoneal lavage fluid cytokine concentration were measured by ELISA for IL-13 **(A)**, GM-CSF **(B)**, and CCL2 **(C)**. Limits of detection were 10pg/ml, 5 pg/ml and 20 pg/ml respectively. Representative flow cytometry gating strategy for identification of lymphocytes (FSC^lo^SSC^lo^), macrophages (SSC^int/hi^F4/80^+^CD11b^+^) and eosinophils (SSC^int/hi^SIGLECF^+^CD117^—^) **(D)**. *Il13* mRNA expression in sorted eosinophils **(E)** and CD19^+^, CD4^+^, CD3^+^ and macrophages **(F)** 3 h after IL-33 or saline treatment was analyzed by ddPCR and expressed as normalized specific abundance (NSA) per 10^3^ cells **(E, F)**. Statistical significance was determined using one-way ANOVA **(A–C, F)** or unpaired t-test **(E)**. *p<0.05, **p<0.01, ***p<0.001, ****p<0.0001.

To examine potential mechanisms by which IL-33 might enhance efferocytosis in macrophage populations, peritoneal lavage fluid samples from IL-33 and saline treated animals at 3h, 6h, 24h, and 48h were assayed for the presence of several cytokines, chemokines, and growth factors. IL-33-treated mice at 3h and 6h post IL-33 injection had significantly increased IL-13 (**Figure 4A**), and GM-CSF (**Figure 4B**) concentrations in lavage fluid compared to saline treated control animals. CCL2 was significantly increased in lavage samples at 3h but not at 6h post IL-33 treatment (**Figure 4C**). This cytokine response was rapid and transient. None of the cytokines assayed were elevated in lavage samples taken 24h or 48h after IL-33 administration (data not shown). IL-4, IL-10, and CCL5 were undetectable in the lavage fluid and no changes were observed in M-CSF (IL-33 vs. saline, 3h p=0.19, 6h p=0.23) and VEGF-A (IL-33 vs. saline, 3h p=0.27, 6h p=0.58) content between conditions (data not shown n=5 per group).

### Granulocytes, not lymphocytes, are the substantial early contributor of IL-13, following IL-33 treatment

IL-13, GM-CSF, and CCL2 are all products of peritoneal granulocytes, but the latter two products are not normally associated with early T cell activation. To better define the potential sources of IL-13 following peritoneal IL-33 injection, mouse peritoneal lavage cells were separated into granulocyte-enriched and granulocyte-depleted populations by Ficoll-Hypaque separation. The fractions were then treated with IL-33 *in vitro*, and the production and release of IL-13 into the supernatant were assessed at early time points by ELISA. Notably, as early as one hour following IL-33 treatment, the granulocyte-enriched fraction of cells produced approximately twice as much IL-13 on a per cell basis (mean 57.4 pg/million cells, n=6) than the granulocyte depleted fraction (23.9 pg/million cells, n=6). To further investigate this issue, macrophages, lymphocytes, and eosinophils were sorted by FACS from IL-33-treated mice 3 h post IL-33 injection (**Figure 4D**) to assess *Il13* expression by qPCR and ddPCR. Three hours after injection of IL-33, significantly increased *Il13* mRNA expression was observed in the eosinophil population (**Figure 4E**) compared to saline-treated mice. *Il13* mRNA was not significantly upregulated in populations of macrophages, CD4^+^, CD8^+^ and CD19^+^ lymphocytes from IL-33-treated animals compared to saline controls (**Figure 4F**). These findings exclude major lymphocyte and macrophage populations as dominant contributors to early IL-13 production in this setting. Yields of viable mast cells post sorting were not sufficient for analysis due to their low frequency in the peritoneal cavity and sensitivity to sorting procedures, which can compromise viability and RNA integrity.

**Figure 5:**
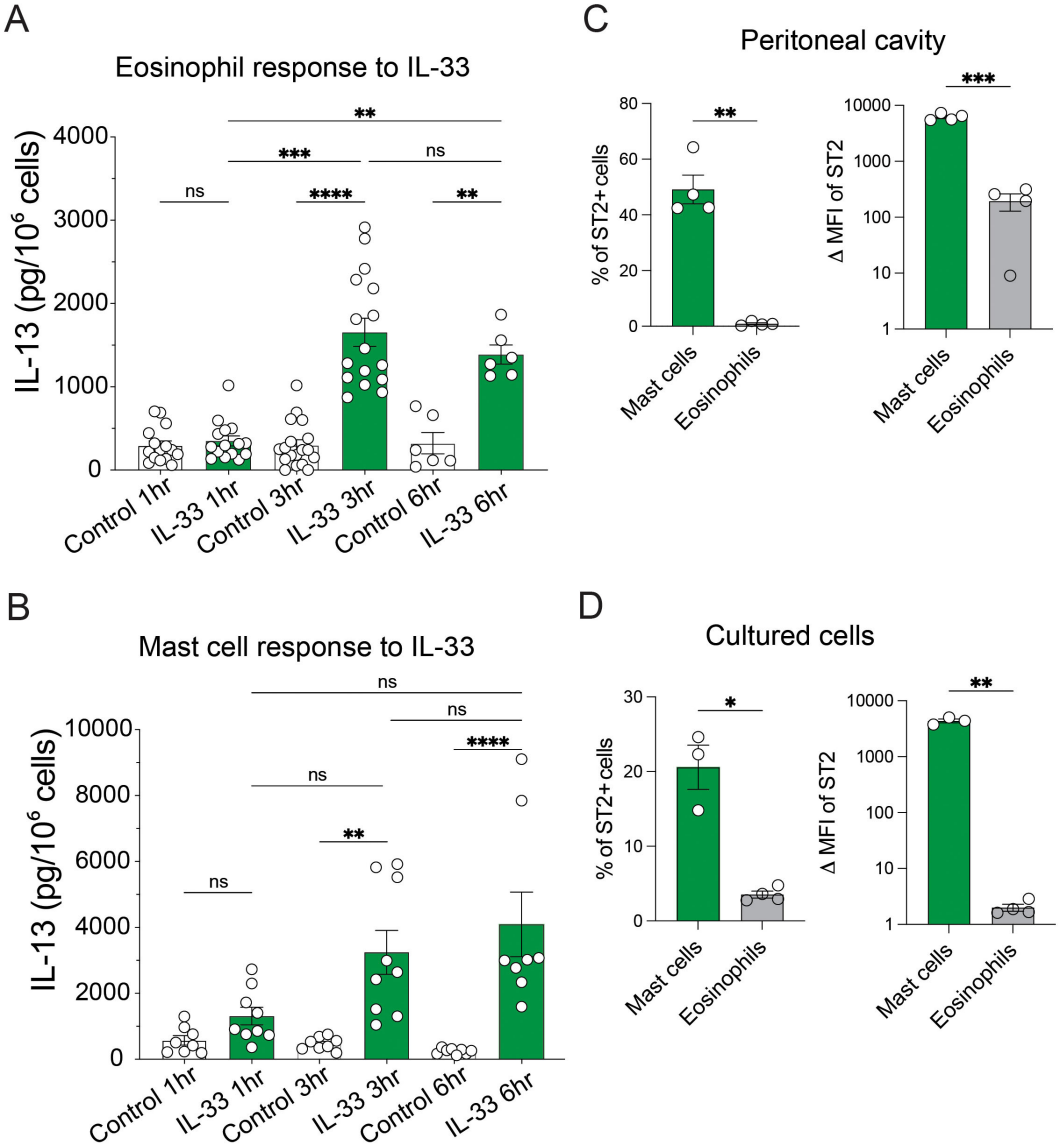
IL-33 rapidly induces IL-13 production and is associated with higher ST2 expression in mast cells compared to eosinophils. Eosinophils **(A)** and mast cells **(B)** were generated from cultures of mouse bone marrow cells and were stimulated with 10 ng/mL of recombinant murine IL-33 for 1, 3, or 6 h. Levels of IL-13 in cell free supernatants were examined by ELISA. Surface expression of ST2 on peritoneal mast cells and eosinophils, shown as percentage of ST2^+^ cells and mean fluorescence intensity (MFI) **(C)**. Surface expression of ST2 on bone marrow–derived mast cells and eosinophils under *in vitro* culture conditions, shown as percentage of ST2^+^ cells and MFI **(D)**. Data represented mean ± SEM from n=4–12 per group. Statistical significance was determined using one-way ANOVA for panels A and B and unpaired two-tailed tests for panels C and **(D)** *P< 0.05, **P< 0.01, ***P< 0.001, ***P< 0.0001; ns, not significant.

Mast cells have been reported to produce IL-13 following IL-33 treatment and were the most frequent ST2 expressing cell type resident in the peritoneal cavity (42, 43). Eosinophils can also serve as a source of IL-13 (44). To analyze pure populations of mast cells and eosinophils for IL-13 production in response to IL-33, we prepared mouse bone marrow-derived mast cells or eosinophils. In separate experiments we examined the IL-13 responses of these two ST2 positive granulocyte populations following IL-33 (10 ng/ml) exposure. Both cultured bone marrow-derived mouse eosinophils (**Figure 5A**) and bone marrow-derived mast cells (**Figure 5B**) produced significant levels of IL-13 protein within 3 hours of IL-33 treatment. This is consistent with higher ST2 expression observed on mast cells compared to eosinophils. These *in vitro* findings may not fully reflect tissue resident cell function and are interpreted alongside *in vivo* data.

**Figure 6:**
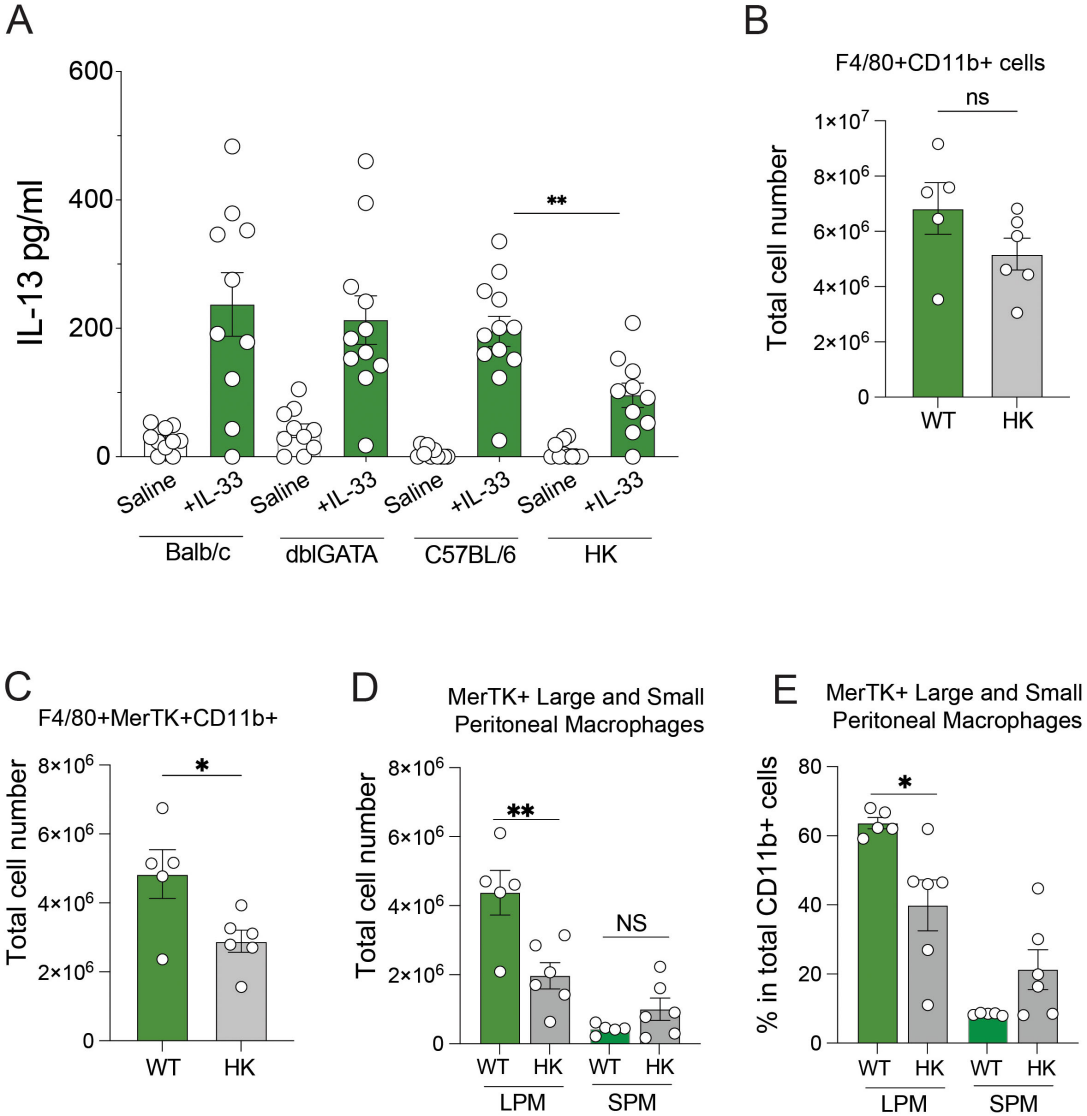
Mast cell deficient (HK) mice have reduced early IL-13 and MerTK response to intraperitoneal IL-33 administration. Female eosinophil-deficient (ΔdblGATA) and mast cell deficient (Cpa3-Cre; Mcl-1^fl/fl, HK), along with wild type controls (BALB/c for ΔdblGATA and mast cell–containing littermates for HK), were injected i.p. with 0.5 ug IL-33 or saline. Peritoneal lavage fluid was collected, using 4ml of saline to flush the peritoneal cavity 3 h or 48 h later. IL-13 levels in peritoneal lavage fluid were measured by ELISA. IL-33 administration significantly increased IL-13 levels in all strains compared to saline controls; however, mast cell–deficient (HK) mice exhibited a significantly reduced IL-13 response compared to wild-type controls **(A)**. Total number of F4/80^+^CD11b^+^ peritoneal macrophages, showing no significant difference between WT and HK mice after 48hr post-injection of IL-33 **(B)**. Total number of F4/80^+^MerTK^+^CD11b^+^ macrophages, demonstrating a significant reduction in mast cell–deficient mice **(C)**. Absolute numbers **(D)** and percentage **(E)** of MerTK^+^ large peritoneal macrophages (LPMs) and small peritoneal macrophages (SPMs). MerTK^+^ LPMs were significantly reduced in HK mice, whereas SPMs were not significantly different. Data are presented as mean ± SEM with individual data points shown. Statistical significance was determined using one-way ANOVA for panel A and unpaired two-tailed tests (or Mann–Whitney, if non-parametric) for panels B–E, as appropriate. **p< 0.01, *p< 0.05; ns, not significant.

To further evaluate the early source of IL-13 in this setting we compared the levels of IL-13 in peritoneal fluid, 3h post-IL-33 administration in mice deficient in either mast cells (Hello Kitty; HK mice) or eosinophils (ΔdblGATA mice). Notably, there was no significant impact of a lack of eosinophils on the IL-13 response (**Figure 6A**) at this time point. The percentage of MerTK-positive cells was also not significantly altered as a result of eosinophil deficiency. However, the absence of mast cells was associated with a significant reduction in IL-13 production in response to IL-33 (**Figure 6A**) confirming mast cells as a substantial early source of IL-13 in response to IL-33 administration in the peritoneal cavity.

To determine whether reduced IL-13 levels in mast cell–deficient mice impact macrophage phenotype, we assessed MerTK expression on peritoneal macrophages. While the total number of F4/80^+^CD11b^+^ macrophages was not significantly altered between WT and mast cell–deficient mice (**Figure 6**), the frequency and absolute number of MerTK^+^ macrophages were significantly reduced in mast cell–deficient mice (**Figure 6**). Further stratification of macrophage subsets revealed that this reduction was primarily driven by LPMs, where MerTK^+^ LPMs number and percentages were significantly decreased in mast cell–deficient mice compared to WT controls (**Figure 6D**). In contrast, SPMs did not show a significant difference in MerTK expression between groups.

Together, these data indicate that mast cells contribute to the maintenance of MerTK expression on macrophages, particularly within the LPM compartment, consistent with a role for mast cell–derived signals, including IL-13, in promoting macrophage efferocytic potential. This supports a functional link between mast cell-derived signals and macrophage phenotype.

## Discussion

Resolution of inflammation is crucial to return tissues to homeostasis. Efferocytosis by macrophages is an important step in this process (10). IL-33 release after cell injury or ongoing production has a number of impacts related to tissue restitution and host defense (20, 37). IL-33 also plays a useful role in providing signals that regulate monocyte/macrophage differentiation and promote efferocytosis (20, 45, 46). In this study, we demonstrate that the administration of IL-33 alone, in the absence of other damage associated stimuli, increases peritoneal MerTK^+^ macrophage populations (**Figure 1D**) and efferocytosis activity (**Figures 2B, G**) following local upregulation of IL-13 protein production (**Figure 4A**) and mRNA expression (**Figures 4E, F**). These findings indicate that IL-33 plays a role in promoting resolution pathways early after tissue damage. IL-13 has previously been shown to increase macrophage expression of MerTK and efferocytosis ability *in vitro* (20–22) and *in vivo* in peritonitis and acute lung injury (21) resulting in macrophage populations with tissue repair phenotypes. IL-13 also enhanced efferocytosis in the context of atherosclerosis, via an IL-10 dependent mechanism (21). Given the critical importance of efferocytosis in tissue repair and inflammation resolution, reports of multiple pathways to induce such activity of macrophages in different tissue settings are not unexpected. This could include distinct short term and longer term cellular and mediator responses.

Epithelial-derived alarmins such as IL-33, TSLP, and IL-25 are rapidly released upon tissue injury or infection in barrier organs, including the lung, intestine, and peritoneal cavity, and can exert either pathogenic or protective effects depending on the tissue site and type of inflammation (47, 48). Our present findings add to this knowledge by showing that intraperitoneal IL-33 administration alone can modify peritoneal leukocyte composition, increasing the local numbers of both large and small peritoneal macrophages, expanding MerTK^+^ macrophages, and enhancing efferocytosis. Mechanistically, IL-33 elicited a rapid and transient increase in IL-13, GM-CSF, and CCL2 in the peritoneal microenvironment, with mast cells identified as the predominant early source of IL-13. To define the early cellular sources of IL-13, we focused on the 3 h time point, which captures the initial period of cytokine induction and minimizes confounding effects from other immune cell recruitment or expansion. While IL-13 protein kinetics were assessed at both 3 h and 6 h, identification of cellular contributors required analysis at the earliest time point to determine primary sources of IL-13 production. This supports a model in which IL-33 initially enhances macrophage efferocytic function through mast cell (and eosinophil)-derived cytokine production.

Importantly, our data identify mast cells as a substantial early source of IL-13 following IL-33 administration. Given that mast cells are long-lived tissue-resident cells, their increased contribution to IL-13 following IL-33 stimulation likely reflects enhanced cytokine production by pre-existing mast cells rather than rapid recruitment or expansion. Prior studies have established a strong mechanistic link between type 2 cytokines and macrophage efferocytosis, demonstrating that IL-4 or IL-13 promotes macrophage-mediated apoptotic cell clearance and tissue repair (20), and that type 2 immune signaling enhances pro-resolving macrophage functions (21, 22). Together, these findings support that IL-33-induced IL-13 contributes to macrophage phenotypic programming and efferocytic capacity. IL-33 has been shown previously to induce IL-13 production by several types of leukocytes and mast cells (28, 30–33). Eosinophils (**Figure 4E**) demonstrated substantial increases in *Il13* mRNA early after IL-33 treatment. IL-33 has previously been shown to promote IL-13 production by eosinophils that contribute to airway inflammation (49). However, the complete absence of eosinophils in ΔdblGATA mice did not reduce the production of IL-13 in response to IL-33 (**Figure 6**). Notably, the preservation of IL-13 production in eosinophil-deficient mice at this early time point further supports the conclusion that eosinophils are not a major contributor to the initial IL-33–driven IL-13 response. These findings reinforce a dominant role for mast cells in mediating the rapid IL-13 increase observed following IL-33 stimulation. As this analysis focuses on an early time point, contribution from other ST2^+^ populations at later stages cannot be excluded. However, given the dynamic nature of type 2 immune responses, it remains possible that eosinophils or other ST2^+^ cell populations contribute to IL-13 production at later time points. Future studies examining the temporal dynamics of IL-13 production will be important to fully delineate these contributions. In our study, lymphocytes and macrophages did not demonstrate enhanced *Il13* mRNA signals early after IL-33 administration in the peritoneal cavity (**Figure 4F**), despite literature evidence suggesting a role for these cells, primarily in more chronic settings (21, 29, 50–52). We observed IL-13 protein in the peritoneal cavity as early as 3h after IL-33 administration (**Figure 4A**), suggesting IL-13 may be pre-formed or rapidly produced. Eosinophils, mast cells and basophils have been reported to constitutively express IL-13 mRNA to allow for rapid cytokine production upon stimulation (44). Given the upregulation of *Il13* mRNA that was observed in eosinophils early after IL-33 treatment in the peritoneal cavity (**Figure 4E**) we considered the possibility that IL-13 was pre-formed in these cells. However, in studies of cultured eosinophils, early IL-13 production and secretion in response to IL-33 was associated, with a concomitant increase in cell-associated IL-13 at 3hrs (**Figure 5**). While other cell populations have been shown to produce IL-13 in response to IL-33 (53), early production of IL-13 by mast cells and eosinophils and the potential role of this pathway in efferocytosis regulation has not been widely considered. In light of increasing use of biologics that limit the action of IL-13 in allergic disease (54), there may be a need to further evaluate the impact of clinical IL-13 blockade on efferocytotic responses.

IL-33 has been previously associated with alternatively activated macrophage phenotypes *in vivo* that promote resolution of chronic inflammation (50, 55, 56). We demonstrate that MerTK^+^ macrophage number, as well as efferocytosis, were increased in response to IL-33. Although IL-33 treatment increased overall efferocytosis, normalization to total CD11b^+^F4/80^+^ macrophage numbers revealed no significant difference in per-cell efferocytic capacity. This suggests that the enhanced efferocytosis is largely driven by increased macrophage abundance. However, as IL-33 also promotes macrophage recruitment, the influx of additional cells may mask functional changes in resident populations, making it difficult to distinguish the relative contributions of resident versus recruited macrophages. This is the first study in which IL-33 has been directly linked to efferocytosis as a mechanism by which it may aid in resolving inflammation. In contrast to these data, a study using macrophages isolated from women with recurrent spontaneous abortion and cultured *in vitro* had predominantly “M1” macrophage populations that enhanced efferocytosis when IL-33 was inhibited (57). Such *in vitro* analysis of macrophages in isolation from the signals provided by the granulocyte populations in response to IL-33 may not fully represent the complex signaling in tissue microenvironments involving intermediate cell types and mediators.

Macrophages in the peritoneal cavity are crucial facilitators of wound repair within local organs (58, 59). Fat-associated lymphoid clusters, or milky spots, have been shown to be important sources of IL-33 in the peritoneal cavity (60), as have mesothelial cells in the peritoneum (61). Upon tissue damage in the peritoneal cavity, these cells release IL-33 into the tissue microenvironment (61). The findings of the current study further identify a novel mechanism by which IL-33 may be exerting its effects in the context of peritoneal inflammation (56) or ovarian cancer metastasis (62). Clinical evidence has shown that increased presence of the soluble IL-33 receptor, sST2, in the plasma of patients with allergic airway disease, systemic lupus erythematosus, and cardiovascular disease (63–65) is predictive of adverse outcomes. Efferocytosis has important impacts in each of these disease settings (16, 66–70).

Together, IL-33 administration increased peritoneal macrophage numbers (**Figure 1A**) and induced IL-13, GM-CSF and CCL2 in lavage fluid (**Figure 4**), mediators known to support myeloid recruitment and survival. All cytokines can be produced by mast cells, and early responses differed between mast cell–deficient and mast cell–sufficient mice, indicating that mast cells are a key contributor to IL-13 production in the peritoneal microenvironment. Importantly, IL-33 promoted an IL-13–rich environment that enhanced macrophage efferocytosis of apoptotic neutrophils. This mechanism aligns with previous evidence linking IL-33 to macrophage-mediated resolution of inflammation. For example, mast cell–derived IL-13 has been shown to drive alternatively activated, pro-repair macrophage phenotypes (55, 71). While IL-33 does not directly induce alternative activation, its capacity to elicit IL-13 and IL-6 from granulocytes provides a pathway by which macrophages acquire efferocytosis functions.

**Figure 7:**
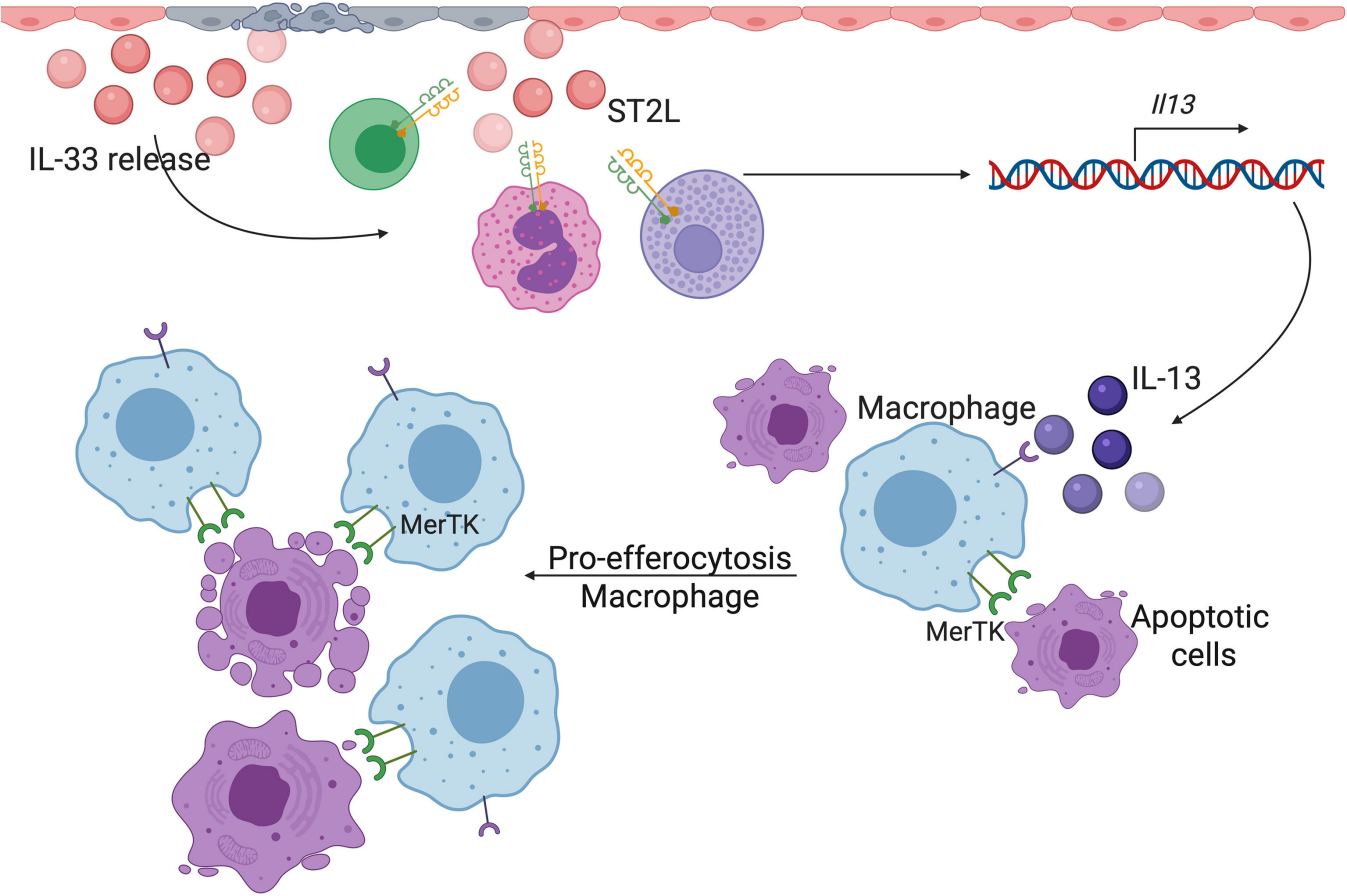
Mechanism by which IL-33 promotes efferocytosis in the peritoneal cavity. IL-33 released as a damage-associated molecular pattern (DAMP) activates local leukocyte populations after tissue damage. IL-33 engages ST2-expressinggranulocytes, including mast cells and eosinophils, triggering rapid induction of *Il13* transcription and secretion of IL-13. This early, localized increase in IL-13 acts on macrophages to enhance MerTK expression, promoting a pro-efferocytotic phenotype. MerTK^+^ macrophages efficiently engulf apoptotic cells, thereby facilitating removal of apoptotic cells, preventing secondary necrosis, and further limiting inflammatory cell death.

In conclusion, the current study shows that IL-33 promotes efferocytosis in the peritoneal cavity by macrophages. This is mediated by the local and rapid induction of IL-13 production, primarily by granulocytes with mast cells being a key source (**Figure 7**). Our work identifies a novel role for IL-33 to promote macrophage efferocytosis and subsequently resolution of inflammation, which could be targeted in both acute and chronic inflammatory settings.

## Materials and methods

### Experimental animals

All mouse experiments were approved by the Dalhousie University Committee on Laboratory Animals (Protocol number 19-031). In IL-33 peritoneal efferocytosis experiments, female mice were used between 6–8 weeks of age. C57BL/6 mice were obtained from Charles River Laboratories (Montreal, Canada). ΔdblGATA mice and BALB/c controls were obtained from Jackson Laboratories (Bar Harbor, ME). Hello Kitty (HK; Cpa3-Cre; Mcl-1^fl/fl^) mice were obtained from Dr. S. Galli and Dr. M Tsai (Stanford University, Stanford, CA).

### Thymocyte isolation and induction of apoptosis

For each efferocytosis experiment, female C57BL/6 mice aged 4–6 weeks were euthanized and dissected to remove the thymus. Thymocytes were isolated and stained with 1μM carboxyfluorescein succinimidyl ester (CFSE) (ThermoFisher, Waltham, MA) prior to induction of apoptosis by incubation in 1 μM dexamethasone in RPMI (ThermoFisher) + 10% FBS (ThermoFisher) for 3 hrs at 37°C in 5% CO_2_. Thymocytes were washed and resuspended in sterile saline prior to injection. To confirm apoptosis by flow cytometry, thymocytes that were not CFSE-labeled were stained with viability dye (efluor506, ThermoFisher) and Annexin V (ThermoFisher) to confirm induction of apoptosis (30-40% Annexin V+; **Supplementary Figure S1D**). The injected population was enriched for early apoptotic cells with minimal necrotic contamination (**Supplementary Table 1**).

### Free mitochondria isolation

Free mitochondria were isolated from C57BL/6 mouse liver using mitochondria isolation kits (ThermoFisher) according to the manufacturer’s instructions. Isolated mitochondrial pellets were suspended in HBSS and treated with Halt Protease Inhibitor Cocktail (ThermoFisher). Protein concentrations of free mitochondria were determined by Bradford Protein Assay (Bio-Rad, Hercules, CA).

### Animal experiments

Mice were injected intraperitoneally (i.p.) with 0.5 μg IL-33 (BioLegend, San Diego, CA) in 100 μL saline, 5 μg free mitochondria in 100 μL saline, a combination of 0.5 μg IL-33 and 5 μg free mitochondria in 100 μL saline, or with 100 μL saline alone. Animals were housed for 24-48h prior to harvesting peritoneal lavage. In efferocytosis experiments, mice were injected i.p. with 100 μL saline or 0.5 μg IL-33 in 100 μL saline and housed for 48hrs prior to i.p. injection of 2.5 x 10^7^ CFSE-labeled thymocytes. After 30 minutes, mice were sacrificed. Peritoneal lavage was conducted with an initial 750 μL volume for protein analysis and subsequent 3 mL volume for cell collection in 0.5% bovine serum albumin (ThermoFisher), and 5 mM EDTA (Invitrogen) in 1x PBS (Wisent Inc.). Cells were washed and counted. Single cell suspensions were then processed for flow cytometry.

### Bone marrow eosinophils and mast cells

The bone marrow was flushed from the tibia of 8-week-old male C57BL/6 mice using RPMI 10% FBS plus penicillin/streptomycin then passed through a 40 μm cell strainer. The red blood cells were lysed using ammonium chloride buffer, washed with excess RPMI 10% FBS, then the cell pellet resuspended at 1x10^6^ cells/ml in BM media (RPMI, 25 mM HEPES, 20% FBS, P/S, NEAA, 50 μM beta-mercaptoethanol, 2 mM glutamine and sodium pyruvate) supplemented with 100 ng/ml mouse GM-CSF (BioLegend), 10ng/ml mouse IL-5 (BioLegend) and 100 ng/ml mouse FLT-3 ligand (Peprotech, Cranbury, NJ). The suspension cells were harvested and replated in fresh media on days 4 and 8, then the cultures were tested at day 11 for eosinophil purity by Diffquik stain (Polysciences Inc., Warrington, PA), and FACS staining for Siglec-F.

Bone marrow-derived mast cells were cultured as described previously (72). Bone marrow cells were cultured in BM media supplemented 200 nM prostaglandin E2 (PGE2) (Sigma-Aldrich, St. Louis, MO), and 15% WEHI 3B cell culture supernatant as a source of IL-3. Cells were replenished with fresh media every 3 days. After 5–6 weeks, mast cells purity was assessed by flow cytometry (checked CD117 expression). Bone marrow-derived mast cells and eosinophils were washed and resuspended in BM media at 1.25x10^6^ cells/ml with/without 10 ng/ml of murine IL-33. Equal volumes of each fraction were aliquoted into a 96 well plate. The supernatants and cell pellet were harvested at 1, 3 and 6 hours and tested for mouse IL-13 by ELISA (PeproTech).

### FACS staining and analysis

Single cell suspensions were stained with fixable viability dye efluor506, and antibodies from BD Biosciences (Franklin Lakes, NJ) antibodies CD11b BV650 (M1/70), Siglec-F PE-CF594 (E50-2440), CD117 BV421 (2B8), CD19 BV605 (1D3), BioLegend CD163 APC (S150491), CD80 biotin (16-10A1), CCR2 FITC (SA203G11), MerTK PE (21310C42), CX3CR1 PerCP-Cy5.5 (SA1011F11), CD206 BV421 (C068C2), Ly6G biotin (1A8), CD4 Alexa Fluor 700 (GK1.5), eBioscience ST2 APC (RMST2-2), FcϵR1 PerCP-efluor710 (Mar-1), Invitrogen Ly6C APC-efluor780 (HK1.4), F4/80 PE-Cy7 (BMB), MHCII Alexa Fluor 700 (M51114.15.2), NK1.1 FITC (PK136). Where biotinylated antibodies were used, streptavidin-BV785 (BioLegend) was subsequently used for visualization. Stained peritoneal cells were acquired for analysis using a BD LSRFortessa flow cytometer (BD Biosciences). Data were analyzed using FlowJo software (Ashland, OR), and macrophages were gated on based on CD11b^+^ and F4/80^hi/lo^ as initial gating.

### Cell sort experiments

Mice were injected i.p. with 0.5 μg IL-33 in 100 μL saline or 100 μL saline alone and housed for 3h prior to sacrifice and necropsy as indicated above. Cells were stained with antibodies from BD Biosciences (Siglec-F PE-CF594), BioLegend (F4/80 PerCP, CD11b PE-Cy7) and eBioscience (CD117 FITC) for macrophages, lymphocytes and eosinophils. Cells were sorted on a FACSAria III flow cytometer into Qiazol (Qiagen, Toronto, Canada) for RNA isolation.

### RNA isolation, reverse transcription, quantitative PCR (qPCR) and droplet digital PCR (ddPCR)

Cells were resuspended in Qiazol and chloroform separation was used to remove protein components from aqueous phase containing nucleic acids. RNA was then isolated from this aqueous phase according to manufacturer’s instruction (RNA isolation kit, Qiagen). A standard quantity of total RNA was reverse-transcribed (Qiagen Quantitect kit). The transcript levels for *Il13* were assessed in macrophages and lymphocytes by SYBRgreen qPCR (Bio-Rad) and in eosinophils by Evagreen ddPCR (Bio-Rad) by using commercially validated primer sets (Bio-Rad and Qiagen) and normalized against *Tbp* and *Gusb* transcript levels. From qPCR data, gene expression values were determined using the formula Δ C_q_= C_q gene of interest_ – C_q Geomean of reference genes_, and normalized expression was then calculated as 2^Δ Cq^. From ddPCR data, normalized standard amount (NSA) was determined by the following formula:


$$\text{NSA}=\frac{\text{Concentration of GOI}\times \text{Dilution factor}}{{\left(\left\{\text{Reference Gene A}\times \text{Dilution factor}\right\}+\left\{\text{Reference Gene B}\times \text{Dilution factor}\right\}\right)}^{1/2}}$$

### Immunoassays

Peritoneal lavage fluid was assayed for cytokines, chemokines and growth factors of interest by Luminex assay. The Luminex assay was conducted according to the manufacturer’s instructions with antibodies specific for IL-4, IL-10, IL-13, GM-CSF, M-CSF, CCL2, CCL5 and VEGF was purchased from R&D Systems (Minneapolis, MN). The samples were read using a Bio-Rad Bio-Plex 2 (Bio-Rad). IL-13 was measured using a commercial ELISA assay kit (R&D systems).

### Statistical analyses

D’Agostino-Pearson normality tests were conducted to determine the appropriate statistical approach. Unpaired students t-tests and ordinary one-way ANOVA were conducted to examine statistical significance between groups. To assess whether IL-33 responses differed between sexes, two-way ANOVA was performed using sex and treatment as factors, including the sex × treatment interaction term. A significant interaction term was interpreted as evidence that IL-33-induced responses differed between male and female mice. Outliers were removed based on ROUT outlier tests of neutrophil and overall cell numbers within each experimental group. All tests were conducted with GraphPad Prism (GraphPad Software Inc., La Jolla, CA).

## Data Availability

The raw data supporting the conclusions of this article will be made available by the authors, without undue reservation.
