# Supplementary material for: IL-33 promotes efferocytosis by peritoneal macrophages by a mechanism associated with rapid granulocyte IL-13 production
Source: Front Immunol. 2026 Jul 22;17:1802300. doi: 10.3389/fimmu.2026.1802300 (PMC13437444; doi:10.3389/fimmu.2026.1802300)
Supplement: Supplementary file 1 [file Table1.docx]

| % | 0.1uM Dex | 1uM Dex | 10uM Dex | 100uM Dex | D10f |
| --- | --- | --- | --- | --- | --- |
| 3h - early apop | 25 | 28.8 | 22.5 | 28.4 | 10.1 |
| 3h - late apop | 3.55 | 3.2 | 3.5 | 2.83 | 1.95 |
| 3h - necrotic | 0.037 | 0.058 | 0.34 | 0.013 | 0.011 |
| 6h - early apop | 37.6 | 43.5 | 43.2 | 45.8 | 15.4 |
| 6h - late apop | 11.4 | 11.1 | 12 | 11.8 | 4.6 |
| 6h - necrotic | 1 | 0.83 | 0.88 | 0.72 | 0.11 |
|  | 0.2uM Camptothecin | 2uM Camptothecin | 20uM Camptothecin | 200uM Camptothecin |  |
| 20h - early apop | 11.3 | 15.6 | 25.9 | 18.3 |  |
| 20h - late apop | 32.4 | 37.1 | 50.3 | 56.7 |  |
| 20h - necrotic | 0.55 | 0.53 | 0.58 | 3.82 |  |
|  | Plain DMEM | 0.01uM Dex | 0.1uM Dex | 1uM Dex |  |
| 20h - early apop | 0.026 | 0.072 | 0.013 | 0.005 |  |
| 20h - late apop | 3.46 | 2.11 | 5.51 | 2.08 |  |
| 20h - necrotic | 20.7 | 32.7 | 82 | 92.1 |  |

**Supplementary Table 1. Flow cytometric characterization of apoptotic thymocyte preparations.**
Thymocytes were treated with dexamethasone for 3 h and stained with Annexin V and viability dye. Percentages of early apoptotic, late apoptotic, viable, and necrotic cells are shown. The injected population was enriched for early apoptotic cells with minimal necrotic contamination.
