# Supplementary material for: IL-33 promotes efferocytosis by peritoneal macrophages by a mechanism associated with rapid granulocyte IL-13 production
Source: Front Immunol. 2026 Jul 22;17:1802300. doi: 10.3389/fimmu.2026.1802300 (PMC13437444; doi:10.3389/fimmu.2026.1802300)
Supplement: Supplementary file 2 [file Table2.docx]

**Supplementary Figure Legends**

**Supplementary Figure 1. Gating strategies to identify leukocyte populations.**

Macrophages were gated first on CD11b^+^ cells, then F4/80^+^ cells. Large peritoneal macrophages were identified as F4/80^hi^CD11b^+^ and small peritoneal macrophages were identified as F4/80^lo^CD11b^+^. Within each macrophage gate, MerTK^+^CD11b^+^ macrophages were also identified. Monocytes were classified as inflammatory (CCR2^+^Ly6C^+^) or patrolling (CX3CR1^+^Ly6C^lo^) in the peritoneal cavity from overall live cells (A). Lymphocytes were identified as FSC lo SSC lo cells and fixable viability dye negative. T cells were identified as CD3^+^ CD19^—^, B cells were identified as CD19^+^CD3^—^, and NK cells were identified as NK1.1^+^CD3^—^ (B) Granulocytes and ILC2s were identified as fixable viability dye negative cells. CD117^+^ST2^+^ cells were classified as mast cells or ILC2s based on FcεR1 gating. Eosinophils were identified as Siglec-F^+^ Ly6G^—^ and further CD117^—^. Neutrophils were identified as Ly6C^+^Ly6G^+^ cells. (C). Efficiency of apoptosis induction by dexamethasone treatment was determined by AnnexinV and Viability staining to identify primary apoptotic (AnnexinV^+^Viability^—^) and secondary apoptotic (AnnexinV^+^Viability^—^). If >10% of cells were necrotic (AnnexinV^—^Viability^+^), cells were not administered (D). Efferocytosis activity was assessed by gating on F4/80^+^CFSE^+^ cells compared to total CFSE^+^ signal (E).

**Supplementary Figure 2. Male mice respond to IL-33 differently than female mice.**

Male mice were given IL-33 (n=8) or saline (n=8) i.p. for 48h prior to sacrifice and lavage. Total numbers are represented as mean ± SEM. Leukocyte populations were assessed by flow cytometry. Macrophages were assessed as F4/80^+^CD11b^+^ cells (A), LPMs as F4/80^hi^CD11b^+^ cells (B), SPMs as F4/80^lo^CD11b^+^ cells (C), and MerTK^+^ macrophages as F4/80^+^MerTK^+^CD11b^+^ cells (D). Patrolling monocytes (E), and inflammatory monocytes (F) were also assessed in the myeloid compartment. In the lymphocyte compartment (G), total T cells (H), B cells (I), NK cells (J), and ILC2s (N) were examined. In the granulocyte/myeloid compartment, eosinophils (K), neutrophils (L), and mast cells (M) were assessed. Total peritoneal cells were counted from lavage fluid (O). Statistical significance was determined using an unpaired t-test. ** p<0.01.

**Supplementary Figure 3. The lymphocyte and granulocyte compartment in female mice after experimental treatment.**

Total numbers are represented as mean ± SEM. Leukocyte populations were assessed by flow cytometry. Leukocytes from female mice treated i.p. with IL-33 for 48h were assessed for ST2^+^ cells (A). In the myeloid compartment, MerTK^+^ LPM (B), and SPM (C) were examined. In the lymphocyte compartment total T cells (D), total B cells (E), and NK cells (F) were identified. In the granulocyte/myeloid compartment, neutrophils (G), inflammatory monocytes (H), and patrolling monocytes (I) were assessed. Leukocytes from female mice treated i.p with IL-33 for 48h, and then with CFSE-labeled apoptotic thymocytes for 30 minutes were assessed for ST2^+^ cells compared to controls (J). MerTK^+^ LPM (K), and SPM (L) populations were also examined. In the lymphocyte compartment, total T cells (M), and total B cells (N) were identified, and in the granulocyte/monocyte compartment neutrophil populations were assessed (O). Statistical significance was determined using an unpaired t-test. *p < 0.05, **p < 0.01, ***p < 0.001, ****p < 0.0001.

### ****Supplementary Figure S4. IL-33 does not enhance per-cell efferocytic capacity or apoptotic cell uptake by macrophages.****

### Data are derived from the same mice and experimental conditions shown in Figure 2. Female C57BL/6 mice were injected intraperitoneally with IL-33 (0.5 μg) or saline and assessed in the presence of CFSE-labeled apoptotic thymocytes. Percentage of F4/80⁺CD11b⁺ macrophages that had engulfed apoptotic cells (CFSE⁺) normalized to total macrophage numbers, showing no significant difference between IL-33- and saline-treated groups (A). Per-cell phagocytic activity, represented as the ratio of CFSE⁺ signal within macrophages, also did not differ significantly between conditions (B). These data indicate that although IL-33 increases overall efferocytosis *in vivo* (data Fig. 2), this effect is primarily driven by increased macrophage abundance rather than enhanced per-cell efferocytic capacity. Data are presented as mean ± SEM with individual data points shown. Statistical significance was determined using unpaired two-tailed tests; ns, not significant.
